# Correction

**DOI:** 10.1080/07853890.2023.2228058

**Published:** 2023-06-24

**Article title:** Protocol for a nested case-control study design for omics investigations in the environmental determinants of islet autoimmunity cohort

**Authors:** Helena Oakey, Lynne C. Giles, Rebecca L. Thomson, Kim-Anh Lê Cao, Pat Ashwood, James D. Brown, Emma J. Knight, Simon C. Barry, Maria E. Craig, Peter G. Colman, Elizabeth A. Davis, Emma E. Hamilton-Williams, Leonard C. Harrison, Aveni Haynes, Ki Wook Kim, Kylie-Ann Mallitt, Kelly McGorm, Grant Morahan, William D. Rawlinson, Richard O. Sinnott, Georgia Soldatos, John M. Wentworth, Jennifer J. Couper, Megan A. S. Penno and the ENDIA Study Group

**Journal:** *Annals of Medicine*

**Bibliometrics:** Volume 55, Number 1

**DOI:** https://doi.org/10.1080/07853890.2023.2198255

This article mistakenly published with wrong Figure 1. The figure below is the correct figure which is now present in the online version.

**Figure 1.**
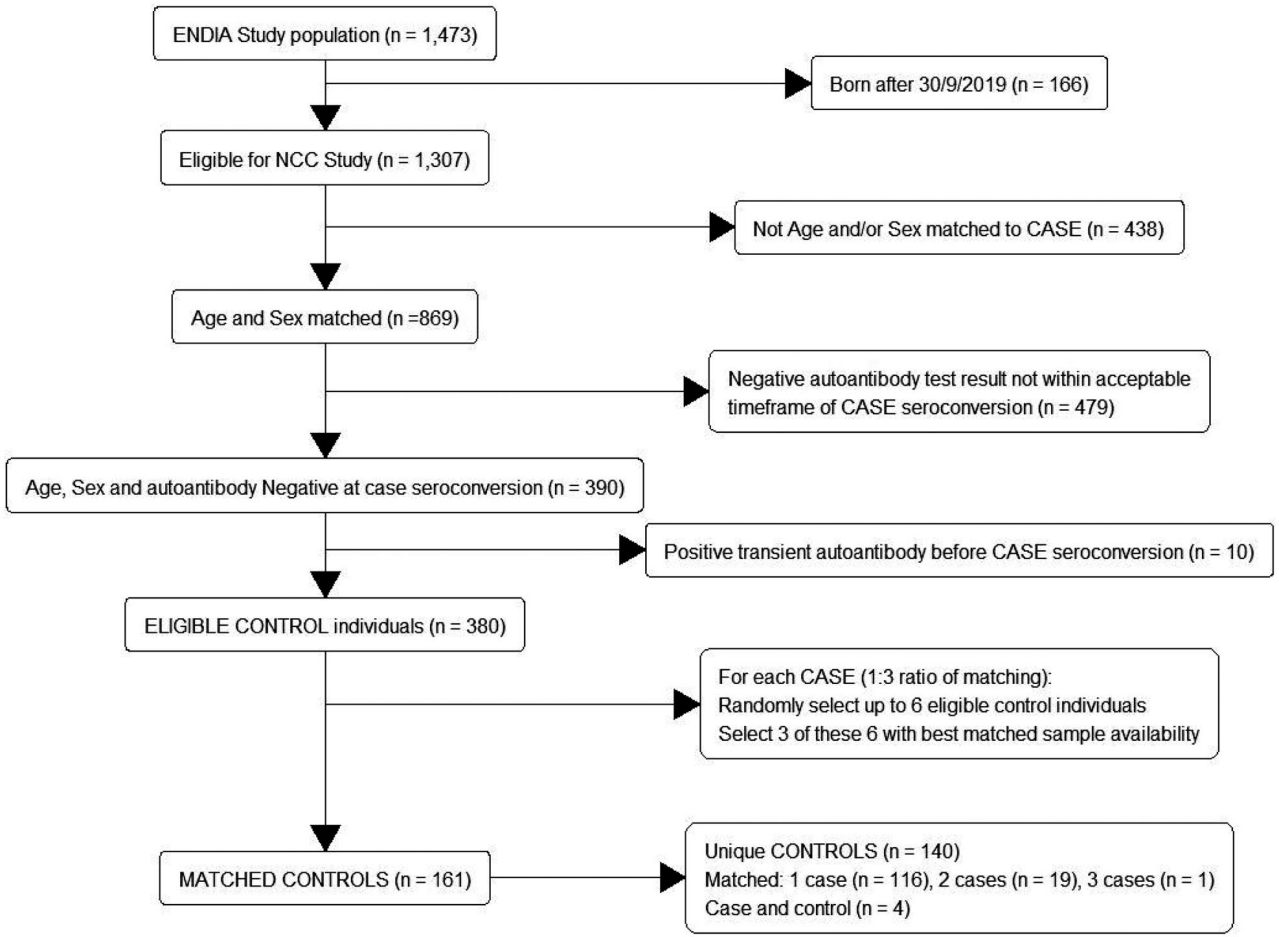
Flow chart for inclusion of children in the nested case control study: matching of controls to cases. *Note:* Cases are eligible to be controls up until their date of onset of persistent IA. Fifty-three cases had three matched controls, one case had two matched controls.

